# Complete and Draft Genome Sequences of the Cruciferous Pathogens Pseudomonas cannabina pv. alisalensis and Pseudomonas syringae pv. maculicola

**DOI:** 10.1128/MRA.00149-21

**Published:** 2021-04-29

**Authors:** Takashi Fujikawa, Yuichi Takikawa, Yasuhiro Inoue

**Affiliations:** a Institute of Fruit Tree and Tea Science, National Agriculture and Food Research Organization (NARO), Tsukuba, Ibaraki, Japan; b Graduate School of Science and Technology, Shizuoka University, Shizuoka, Shizuoka, Japan; c Central Region Agricultural Research Center, NARO, Tsukuba, Ibaraki, Japan; University of Arizona

## Abstract

Pseudomonas cannabina pv. alisalensis and Pseudomonas syringae pv. maculicola cause bacterial leaf blight and bacterial leaf spot of crucifers (Brassicaceae). Both pathogens are threats to the cultivation of cruciferous crops. Here, we sequenced two strains of each pathogen, which will contribute to the development of countermeasures for the above diseases.

## Abstract (precis)

Pseudomonas cannabina pv. alisalensis and P. syringae pv. maculicola cause bacterial leaf blight and bacterial leaf spot of crucifers (Brassicaceae). Both pathogens are threats to the cultivation of cruciferous crops. Here, we sequenced two strains of each pathogen, which will contribute to the development of countermeasures for the above diseases.

## ANNOUNCEMENT

Pseudomonas cannabina pv. alisalensis and Pseudomonas syringae pv. maculicola cause bacterial leaf blight in crucifers and bacterial leaf spot of crucifers, respectively (1). Both pathogens disturb the global production of cruciferous (Brassicaceae) crops. In particular, root-browning symptoms in Japanese radish caused by both pathogens is a dilemma in Japan (2). It is important to elucidate the common pathogenic genes or identify the unique genes for the control of these pathogens. We report the complete genome sequences of *P. cannabina* pv. alisalensis MAFF 301419 and P. syringae pv. maculicola MAFF 302723 (both Japanese radish isolates) and the draft genome sequences of *P. cannabina* pv. alisalensis MAFF 106156 (Chinese cabbage isolate) and P. syringae pv. maculicola SUPP 2206 (turnip isolate).

The MAFF strains were deposited in the NARO Genebank, and the SUPP strain was maintained in the Shizuoka University Plant Pathology Culture Collection. Information about all the strains used in this study (original hosts, locations, and years) is described in our previous paper (2). Each strain was recovered on yeast peptone agar medium from freeze-dried stocks, and these were cultivated in yeast peptone broth at 27°C for 1 day. Then, 1-ml aliquots were used for genomic DNA extraction using a Wizard genomic DNA purification kit (Promega, Madison, WI, USA).

Complete genome sequences were determined using a hybrid approach, combining Nanopore long-read sequencing and DNBseq short-read sequencing (Bioengineering Lab. Co., Ltd., Kanagawa, Japan). For long-read sequencing, a ligation sequencing kit was used (SQK-LSK-109; Oxford Nanopore Technologies Ltd. [ONT], Oxford, UK) without DNA shearing; sequencing was conducted using a GridION X5 system (ONT) on an R9.4.1 flow cell (FLO-MIN106). Long-read sequence data were base called using Guppy v4.0.11+f1071ce (high-accuracy base-calling mode) (3) for MAFF 301419 (36,090 reads with an average length of 14,282 bp; 515 Mbp) and MAFF 302723 (303,374 reads with an average length of 3,279 bp; 995 Mbp). For short-read sequencing, the MGIEasy FS DNA library prep set was used (MGI Tech, Shenzhen, China) according to the manufacturer’s protocol. Subsequently, 2 × 200-bp paired-end sequencing was performed using the DNBSEQ-G400 platform (MGI Tech) according to the manufacturer’s instructions, yielding 5,007,466 (1.0 Gbp) and 5,392,438 (1.1 Gbp) short reads for MAFF 301419 and MAFF 302723, respectively. The reads were assembled *de novo* following a hybrid Nanopore DNBseq approach using Unicycler v0.4.7 (4) and annotated with Prokaryotic Genome Annotation Pipeline (PGAP) v4.3 (5). Default parameters were used for all the above software unless otherwise specified. Chromosomes and plasmids were assembled into single sequences, and we found that both ends of each sequence were overlapped, indicating that these sequences are circular.

The draft genome sequences were determined by the Beijing Genomics Institute (Shenzhen, China) using the HiSeq 4000 platform (San Diego, CA, USA). Short-read sequencing (2 × 100-bp paired-end format) was carried out as described in our previous report (6), yielding 16,287,890 (1.6 Gbp) and 16,481,732 (1.6 Gbp) short reads for MAFF 106156 and SUPP 2206, respectively. The reads were assembled *de novo* using SOAPdenovo v1.05 (7) and annotated with PGAP v4.3. Default parameters were also used for all the above software unless otherwise specified.

Genomic information is listed in Table 1. All the obtained sequences were quality checked using CheckM (8), with 100% completeness. The genome sizes of MAFF 301419 and MAFF 106156 were 6.14 and 6.25 Mbp, with G+C contents of 58.7 and 57.4%, respectively; those of MAFF 302723 and SUPP 2206 were 6.42 and 6.44 Mbp, with G+C contents of 58.3 and 55.9%, respectively. PGAP identified 5,486 to 5,981 genes, including multiple rRNA and tRNA genes. MAFF 301419 and MAFF 302723 contained one and two plasmids, respectively. The genome information obtained in this study will contribute to developing countermeasures for mentioned diseases.

**TABLE 1:** Genome data and accession numbers for the P. cannabina pv. alisalensis and P. syringae pv. maculicola strains

				Genome information						PGAP^*a*^ annotation			Completeness check^*b*^	
Pathogen	Strain	Sequence status	Genome structure	GenBank accession no. (assembly no.)	Genome size (bp)	G+C content (mol%)	No. of contigs	Genome coverage (×)	*N*_50_ (bp)	Total no. of genes	No. of rRNAs	No. of tRNAs	Com (%)	Con (%)
Pseudomonas cannabina pv. alisalensis	MAFF 301419	Complete genome sequence	1 chromosome, 1 plasmid	CP067022, CP067023 (GCF_016599635.1)	6,144,893	58.7	2	247	6,103,677	5,486	17	64	100	0.22
Pseudomonas cannabina pv. alisalensis	MAFF 106156	Draft genome sequence	Undetermined	JAEVFO000000000	6,253,010	57.4	211	260	109,379	5,741	12	57	100	0.61
Pseudomonas syringae pv. maculicola	MAFF 302723	Complete genome sequence	1 chromosome, 2 plasmids	CP067024, CP067025, CP067026 (GCF_016599655.1)	6,419,483	58.3	3	323	6,349,611	5,788	16	64	100	0.38
Pseudomonas syringae pv. maculicola	SUPP 2206	Draft genome sequence	Undetermined	JAEVFP000000000	6,439,699	55.9	231	256	68,797	5,981	8	60	100	0.51

a PGAP, NCBI Prokaryotic Genome Annotation Pipeline.

b The completeness check was performed using CheckM. Com, completeness; con, contamination.

### Data availability.

The complete genome sequences of *P. cannabina* pv. alisalensis MAFF 301419 (accession no. CP067022 and CP067023; assembly no. GCF_016599635.1) and P. syringae pv. maculicola MAFF 302723 (accession no. CP067024, CP067025, and CP067026; assembly no. GCF_016599655.1), as well as the draft genome sequences of *P. cannabina* pv. alisalensis MAFF 106156 (accession no. JAEVFO000000000) and Pseudomonas syringae pv. maculicola SUPP 2206 (accession no. JAEVFP000000000), have been deposited in GenBank. The raw sequencing reads were deposited under the SRA accession no. SRR13295144 and SRR13295143 for MAFF 301419, SRR13295147 and SRR13295146 for MAFF 302723, SRR13295759 for MAFF 106156, and SRR13295745 for SUPP 2206.
